# Perception of clinical management in the child and adolescent population with acute mental health conditions

**DOI:** 10.15649/cuidarte.5110

**Published:** 2026-05-12

**Authors:** Lorena Elizabeth Mondaca Pavié, Edith Elina Rivas Riveros, Catalina Constanza Sepúlveda Rivas

**Affiliations:** 1 Master’s Degree in Nursing with a concentration in Care Management, Universidad de La Frontera. Lecturer, School of Health Sciences, Universidad San Sebastián, Valdivia, Chile. E-mail: lorena.mondaca96@gmail.com Universidad de La Frontera Valdivia Chile lorena.mondaca96@gmail.com; 2 PhD in Nursing, Universidad Andrés Bello. Lecturer, Department of Nursing, School of Medicine, Universidad de La Frontera, Temuco, Chile. E-mail: edith.rivas@ufrontera.cl Universidad Andrés Bello Temuco Chile edith.rivas@ufrontera.cl; 3 PhD in Nursing, Universidad Andrés Bello. Lecturer, School of Medicine, Universidad Santo Tomás, Temuco, Chile. E-mail: catalinasepulvedari@santotomas.cl Universidad Andrés Bello Temuco Chile catalinasepulvedari@santotomas.cl

**Keywords:** Day Care, Medical, Mental Health, Adolescent, Clinical Governance, Centros de Día, Salud Mental, Adolescente, Gestión Clínica, Hospital Dia, Saúde Mental, Adolescente, Governança Clínica

## Abstract

**Introduction:**

Child and Adolescent Day Hospitals stabilize acute mental health conditions through intensive and interdisciplinary interventions. In Chile, there are 12 such facilities, with a shortfall of 73, amid exponential demand growth. This issue coexists with a lack of information and knowledge regarding the care and management provided in these centers. The problem is grounded in Patricia Benner’s theory and E. Morin’s theory of “complex thinking.”

**Objective:**

To reveal the clinical management of children and adolescents enrolled in Child and Adolescent Day Hospitals with severe mental health conditions.

**Materials and Methods:**

A phenomenological study, based on the theoretical framework of Max Van Manen. Twelve in-depth interviews were conducted with a convenience sampling. Data analysis takes a phenomenological approach, identifying units of meaning and categories.

**Results:**

The following meta-categories were identified: 1. Care and management: meso-level, micro-level, and quality; and 2. Challenges related to care: improvement of organizational guidelines, awareness, visibility, and education regarding facilities.

**Discussion:**

Studies reveal centers with similar objectives in mental health, showing greater development in quality processes and in the importance/awareness of the field, alongside specialization in mental health nursing.

**Conclusion:**

The vulnerability and complexity of individuals receiving care are decisive, making community educational awareness and the provision of resources for management and quality of care imperative, alongside an appropriate care model.

## Resumen

**Introducción:**

Los Hospitales de Día Infanto Adolescente estabilizan cuadros agudos de salud mental, por medio de intervenciones intensivas e interdisciplinarias. En Chile, existen 12 dispositivos con una brecha de 73, en un contexto de crecimiento exponencial de la demanda, problema que coexiste con déficit de información y conocimiento respecto a los cuidados y gestiones que se otorgan en estos centros. El problema se apoya en la teoría de Patricia Benner, y la teoría de E. Morin del “Pensamiento complejo”.

**Objetivo:**

Develar la gestión clínica en niños, niñas y adolescentes adscritos a Hospital de día Infanto Adolescentes con diagnósticos de salud mental en condición de gravedad.

**Materiales y métodos:**

Estudio con enfoque fenomenológico, bajo el referente teórico Max Van Manen. Se realizan 12 entrevistas en profundidad con muestreo por conveniencia, el análisis toma actitud fenomenológica, reconociendo unidades de significados y categorías.

**Resultados:**

Se obtienen metacategorías de: 1. Cuidados y gestión: cuidados a nivel de mesogestión, microgestión y calidad y 2. Desafíos en relación con los cuidados: mejora de lineamientos organizacionales, concientización, visualización y educación del dispositivo.

**Discusión:**

Estudios develan centros con similares objetivos en salud mental, observándose mayor desarrollo en los procesos de calidad y en la importancia/concientización del área, coexistiendo con especialización de enfermería en salud mental.

**Conclusiones:**

Es determinante la vulnerabilidad y complejidad de las personas de cuidado, por lo que se hace imperante la concientización educativa en la comunidad y la prestación de recursos para gestión y calidad en la atención en conjunto a un modelo de atención pertinente.

## Resumo

**Introdução:**

Os Hospitais-Dia da Infância e Adolescência estabilizam quadros mentais agudos por meio de intervenções intensivas e interdisciplinares. No Chile, existem 12 dessas unidades, com uma lacuna de 73, em um contexto de demanda crescente exponencialmente. Esse problema coexiste com a falta de informação e conhecimento a respeito do cuidado e manejo oferecidos nesses centros. Essa questão é corroborada pelas teorias de Patricia Benner e pela teoria do "Pensamento Complexo" de E. Morin.

**Objetivo:**

Compreender o manejo clínico em crianças e adolescentes internados em Hospitais-Dia da Infância e Adolescência com diagnósticos de saúde mental em condição grave.

**Materiais e métodos:**

Foi realizado um estudo fenomenológico, baseado no referencial teórico de Max Van Manen. Doze entrevistas em profundidade foram conduzidas utilizando amostragem por conveniência. A análise adotou uma abordagem fenomenológica, identificando unidades de significado e categorias.

**Resultados:**

As seguintes metacategorias foram obtidas: 1. Cuidado e gestão: cuidado nos níveis de mesogestão, microgestão e qualidade; e 2. Desafios relacionados ao cuidado: aprimoramento das diretrizes organizacionais, conscientização, visibilidade e educação sobre o serviço.

**Discussão:**

Os estudos revelam centros com objetivos semelhantes em saúde mental, demonstrando maior desenvolvimento nos processos de qualidade e na importância/conscientização da área, coexistindo com a especialização em enfermagem em saúde mental.

**Conclusões:**

A vulnerabilidade e a complexidade dos usuários do cuidado são fatores determinantes, tornando imperativa a conscientização educativa na comunidade e a disponibilização de recursos para a gestão e a qualidade do cuidado, juntamente com um modelo de cuidado relevante.

## Introduction

Child and Adolescent Day Hospitals (CADHs) provide care for children and adolescents (C&A) aged 10 to 18 years, aiming to stabilize acute mental health conditions through outpatient, intensive care delivered by a multidisciplinary team. They respond to new health strategies in Chile by offering management of risk behaviors, psychosocial and pharmacological treatment, while also providing an alternative to hospitalization, thereby shortening stays in tertiary care hospitals (pediatric or emergency units) or in Psychiatric Intensive Care Units (PICUs). There is a high demand for these services and a shortage of 187 beds^1^-^5^.

In Chile, there are currently only 12 Child Day Hospitals (CDHs), despite the exponential growth in mental health needs, particularly following the consequences of COVID-19. This situation is exacerbated by low awareness of the subject, compounded by significant gaps in financial, human, and material resources, as well as in the quality of care provided. The lack of a management model that provides technical guidance to the healthcare teams could further deepen these gaps and exacerbate these critical issues^5^.

According to the World Health Organization (WHO), one in seven young people aged 10 to 19 has a mental disorder and experiences lower levels of social, psychological, and physical well-being. Depression, anxiety, behavioral disorders, and suicide are the most prevalent conditions, with suicide being the fourth leading cause of death among adolescents aged 15 to 19^6^-^8^. Collectively, some of these conditions had repercussions during the pandemic, including changes in daily routines and increased symptoms of anxiety, fear, and stress. Furthermore, according to the United Nations Children’s Fund (UNICEF), 1 in 7 children has been directly affected by lockdown measures, a quarter have experienced anxiety, and 15% have experienced depression^9^,^10^.

When addressing the population’s demands, it is important to consider the determinants of health as well as the facilitating factors^3^, for the development of a rights-based approach^3^. Consequently, the 2017 Mental Health Plan reveals a higher prevalence of affective and disruptive disorders among children and adolescents with a history of sexual abuse, family psychopathology, exposure to abuse, and single-parent families. Additionally, 69.1% of children and adolescents in child protection services have mental disorders, with 45.3% at risk of suicide and 40% presenting drug dependence^11^,^12^.

These vulnerability factors represent some of the main critical challenges within the Mental Health Network, a situation further exacerbated by the fact that a large proportion of this population resides in residential care facilities under ongoing legal proceedings for the protection of their rights. This hinders network coordination, administrative management, and clinical care due to the absence of stable guardians and high personnel turnover in these institutions. It is worth noting that there is an 88.9% gap in care coverage to meet mental health needs, which is increasing progressively each year^3^.

In patient care, the multidisciplinary clinical team performs interventions^13^ in an environment marked by a lack of infrastructure, equipment, and human resources, as well as inconsistencies in contract terms and a shortage of training, undergraduate education, and continuing education, all of which affect user satisfaction^12^.

Regarding clinical management in mental health facilities, it is hindered by the aforementioned gaps, particularly the lack of a management model aligned with current demands, as the most recent model was created in 2001, which has led to difficulties in standardizing and ensuring quality across centers, especially in CADHs. Clinical management plays an important role and function in mental health, enabling the planning, directing, coordinating, negotiating, monitoring, and evaluating of internal clinical care processes, while identifying areas for improvement to ensure quality in patient care and meet the objectives set forth in the National Mental Health Plan. This could be demonstrated through indicators that enable monitoring and promote continuous improvement, transforming data into relevant information that informs clinical decision-making and even public health policy^14^-^16^.

The complexity of care is extensive; therefore, interventions are conducted with a comprehensive approach that addresses the condition as well as its socioeconomic, cultural, and relational aspects. The complexity of the condition includes its severity, the intensity of the clinical presentation, personal vulnerability, and the community contex^12^.

Empirically, at a Spanish center, the following activities are reported: patient care, coordination, continuing education, supervision, and educational activities^17^. In turn, a study published in CADHs reports that adolescents with psychotic disorders show improvement six months after admission and that educational and social network-based interventions could increase treatment effectiveness and reduce the length of stay in these facilities, thereby enhancing therapeutic benefits^18^.

Another study highlights the clinical team’s capacity and sensitivity to identify warning signs, make timely referrals, and operate within the therapeutic community model^19^ and psychosocial care. The polarization between biomedical and biopsychosocial approaches can significantly influence clinical outcomes for patients and their families^20^. In addition, significant improvements in symptom burden have been reported, along with increased quality of life and high levels of treatment satisfaction. Among the influencing factors are illness severity and the therapeutic relationship, the latter of which is highly valued by both parents and professionals^21^.

The experiences of both users and personnel have also been positive, with particular emphasis on the therapeutic value of the interventions and the strengthening of social connections^22^. From a clinical perspective, there is a reduction in symptoms related to externalizing problems, as well as functional improvements at home, at school, in peer relationships, and in recreational activities^23^. Along the same lines, there is evidence of a significant reduction depressive symptom severity and improved overall functioning. Adolescents report high satisfaction with individual and group treatments, working with their families, and coordination with the school system^24^.

Furthermore, the role of coping is crucial; a more challenging family environment is associated with being less likely to seek support focused on emotional regulation and cognitive restructuring^25^. Similarly, a German study offers an innovative, community-based, and preventive treatment program with a multidisciplinary, group-based approach, including psychotherapy for parents, family therapy, and video-based support for observing interactions^26^. These elements are necessary for change, adherence to treatment, and the development of positive, supportive relationships with peers^27^.

Theoretically, Marjory Gordon’s theory highlights differences in overall functioning between admission (49%) and discharge (81%), noting improvements in illness awareness, sleep hygiene, personal hygiene, and memory, as well as decreases in risk behaviors and increases in coping strategies for stress and anxiety^28^. Similarly, a study conducted in Madrid demonstrated the effectiveness of nursing assessments in identifying potential health issues, thereby contributing significantly to interdisciplinary collaboration and a more effective therapeutic relationship^29^.

In Chile, there is a notable association between mood disorders and schizophrenia and abuse and attachment issues, likely due to the presence of family situations with a history of abuse and rights violations^30^. In Canada, CADHs have demonstrated the effectiveness of intensive, multimodal psychiatric day treatment programs in reducing symptoms, showing significant functional improvement at home, at school, in interpersonal relationships with peers, and during leisure time^31^.

In terms of scale, there is a shortage of 73 healthcare facilities compared to the standard of 85. In Latin America, the prevalence of psychiatric disorders among children and adolescents is 22.5%, with the highest rates seen among children aged 4 to 11. Furthermore, 24.8% of these children have some form of disorder, although less than half of the population requiring medical care actually seeks it^32^. This demand has continued to rise; in 2018, 16.5% of adolescents had some form of mental illness, and 11.5% reported difficulties with sleep hygiene. Similarly, 1 in 4 young people reported feeling sad, discouraged, or depressed during the past month, while 3% reported having suicidal thoughts “always” or “almost always,” particularly among those aged 15 to 19^33^.

The COVID-19 pandemic exacerbated symptoms in children and adolescents with pre-existing psychiatric disorders. Furthermore, the prevalence of anxiety and depressive symptoms among children and adolescents has doubled during the pandemic^9^,^34^, in settings that include therapeutic case managers, providing comprehensive care plans (CCPs) or individualized treatment plans (ITPs), including home-based mental health visits and crisis intervention involving emotional, environmental, and pharmacological support, as well as physical restraint if necessary^35^.

As this subject remains at an early stage of research and development in Chile, the theoretical framework drawn upon is Patricia Benner’s Philosophy of Care^36^, in which practice and theory engage in a dialogue, advancing nursing knowledge through practice, via research, observation, documentation, and the development of practical expertise in clinical work^36^,^37^. This will motivate nursing professionals to identify areas for continuous improvement in this field, which is experiencing increasing demand and in which they play a leading role. It also draws on Edgar Morin’s Theory of Complex Thought, in which the complexity of care seeks answers through a transdisciplinary approach, aiming to establish connections among scientific, philosophical, ideological, and artistic knowledge across various sciences, whether human or natural^38^,^39^, iThis approach integrates different dimensions to address broader-scope issues. For this reason, this study is conducted as a qualitative study with a phenomenological approach inspired by Van Manen, which enables an understanding of lived experience and explores its depth, allowing for an understanding of reality in its natural state.

This study aimed to reveal the perception of clinical management provided to children and adolescents (C&A) with mental health diagnoses who enrolled in Child and Adolescent Day Hospitals (CADHs) within Chile’s public healthcare system. The study was conducted in 2023 within a specialized community mental health facility designed for intensive outpatient care of children and adolescents, whose operations are coordinated by interdisciplinary teams that comprehensively address the biopsychosocial needs of this population.

## Materials and Methods

A qualitative study with a phenomenological approach was conducted, aiming to understand reality in its natural setting by interpreting phenomena based on the meanings they hold for those who experience them, and by exploring that lived experience in depth^40^-^44^.

The study was conducted in 2023 within the context of mental health care for children and adolescents at a healthcare facility of the Chilean public health system. It includes the Child and Adolescent Day Hospital (CADH) and the Department of Mental Health, where interdisciplinary clinical teams and technical advisors at the management level are involved.

The selection of the phenomenological design was motivated by the need to explore the subjective experiences of professionals and managers in these settings and to grasp the meanings constructed around their clinical and managerial practices, particularly in relation to the implementation of asset-based models in mental health.

According to Van Manen, lived experience refers to human experience as perceived through reflective consciousness, to recover the essence of what has been lived. Phenomenological description seeks to express this experience through language, thereby responding to the essence of the phenomenon described^45^.

The phenomenological analysis proposed by Van Manen is structured around three interrelated stages that together form a circular, continuous process of reflection^45^,^46^.

In the **first stage**, data are collected through phenomenological interviews centered on language. This process includes a detailed transcription of each account, which allows for subsequent rigorous data coding^45^.

The **second stage** involves a reiterative reflection on the experience lived. At this stage, general themes are identified from the narrated content and organized into two levels: macro-thematic reflection, which addresses the core meaning of the experience, and micro-thematic reflection, which breaks down the narrative into specific phrases or units of meaning. These units reveal the deeper meaning of the experience being recounted^45^.

The **third stage** corresponds to a descriptive-reflective process that explores the previously identified units of meaning in greater depth. This stage entails an operational integration of meanings through concept maps, illustrating the relationships between the various units and emerging themes^45^.

The target population consisted of personnel working in child and adolescent mental health within a healthcare facility, including clinical teams and managers from the Child and Adolescent Day Hospital (CADH) and the Department of Mental Health. The unit of analysis consisted of accounts from managers and clinical teams at Child and Adolescent Day Hospitals, as well as technical advisors from residential care facilities and supportive housing, and the heads of the Department of Mental Health.

Convenience sampling was used, complemented by snowball sampling^41^,^42^,^47^, until data saturation was reached. The final sample consisted of 12 participants: a nurse, a social worker, a psychologist, an educational psychologist, a child and adolescent psychiatrist, an occupational therapist, a senior nursing technician (SNT), a CADH coordinator, a CADH technical director, three technical advisors from the Department of Mental Health, and a department head.

The study included professionals in leadership roles (such as department heads, technical directors, or technical advisors) in the field of mental health, with at least one year of experience in their position; coordinators of community mental health centers with a minimum of one year of experience; and clinical professionals such as social workers, psychologists, nurses, psychiatrists, occupational therapists, advanced practice nurses, and educational psychologists or special education teachers, who worked in Child and Adolescent or Adult Day Hospitals, with at least one year of experience in those positions.

Personnel from Child and Adolescent Day Hospitals whose duties were limited exclusively to administrative or institutional support tasks (e.g., secretarial personnel, mailroom personnel, assistants) were excluded, as were professionals on extended medical leave for a period exceeding one year.

Data were collected through in-depth interviews using a semi-structured guide designed to facilitate the exploration of participants' lived experiences regarding Child and Adolescent Day Hospitals (CADHs). The interviews were guided by a general leading question: “Could you tell me everything you know about child and adolescent day hospitals based on your experience?”This question allowed the narrative to unfold from a subjective perspective, enabling deeper exploration through flexible follow-up questions that followed the flow of the conversation.

The interviews were conducted in person in a meeting room at the CADH, which ensured privacy and fostered a comfortable and confidential atmosphere. Interviews were scheduled outside participants’ regular work hours, considering their availability and well-being. Each interview lasted approximately one hour.

With the aim of ensuring interpretive fidelity, field notes were taken in tandem with the interviews by a research assistant. All interviews were audio-recorded after each participant provided written informed consent. They were subsequently transcribed manually to ensure greater interpretive fidelity. In line with the principle of confidentiality, personal names and identifying references were replaced with alphanumeric codes in both the transcripts and the reported results.

Data collection continued until theoretical saturation was reached, that is, the point at which the information collected became repetitive and no longer yielded new categories or meanings relevant to the phenomenon under study^40^-^42^.

A theoretical approach consistent with phenomenology was adopted, assuming a reflective stance toward the experience under study, aimed at returning to the original lived experience and its meanings. In this context, the principle of epoché (an ancient Greek term meaning “suspension”) was applied, understood as a deliberate stance of refraining from judgments, theories, or prior knowledge, allowing the experience to manifest itself, without being mediated by preconceived interpretations^43^,^46^,^48^.

Once the transcriptions were completed, the study proceeded with phenomenological analysis based on the second and third stages proposed by Van Manen, as described by Guerrero-Castañeda^45^. In the second stage, units of meaning were identified using open-coding and axial coding, with the support of ATLAS.ti version 23. This software enabled the systematic segmentation of the data into meaningful units, facilitating the thematic and conceptual organization of the analyzed corpus.

Subsequently, macro-thematic reflection was conducted, focusing on core meanings, along with micro-thematic reflection, which delved into key phrases or passages from the accounts. In the third stage, units of meaning were integrated by identifying thematic relationships and, when appropriate, representing them through concept maps or diagrams, thereby fostering a structured understanding of the lived experience^41^,^45^.

Throughout the entire process, the trustworthiness criteria proposed by Guba and Lincoln were applied: credibility, transferability, dependability (consistency), and confirmability (reflexivity)^49^,^50^. With the aim of ensuring the validity of the findings and minimizing potential biases arising from a single source or the researcher’s individual interpretation, triangulation was used. In this study, investigator triangulation was applied, allowing the analysis to be contrasted and enriched from different perspectives, thereby adding rigor, breadth, and depth to the results. Thus, a second researcher independently reviewed and analyzed the data, strengthening the study’s consistency and conformability^51^. Furthermore, ethical principles were upheld in line with the seven requirements proposed by Emanuel et al.^52^: social value, scientific validity, equitable participant selection, favorable risk-benefit ratio, independent review, informed consent, and respect for research participants^52^-^55^. The data collected in their entirety are available for free access and consultation on Mendeley Data^56^.

This study was approved by the Scientific Ethics Committee of the Los Ríos Health Service in Chile, under Protocol No. 241.

## Results

The core of the phenomenon is addressed through 12 in-depth interviews with informants from the clinical, micro-level, and management areas, as well as technical advisors from the CADH, residential care facilities, and supportive housing within the meso-level. All interviewees have more than five years of experience in mental health and are between 29 and 55 years old.

Four main meta-categories emerged, each organized into its respective subcategories, as detailed below:

**1. Meso-level management:** Comprising two subcategories: 1.1 Intersectoral care; 1.2 Care following referral.

**2. Micro-level management:** Comprising 4 subcategories: 2.1. Coordination; 2.2. Intersectoral collaboration; 2.3. Education; 2.4. Community context.

**3. Quality.**

**4. Challenges related to care.**

Each of these is discussed in detail below.

**Figure 1:**
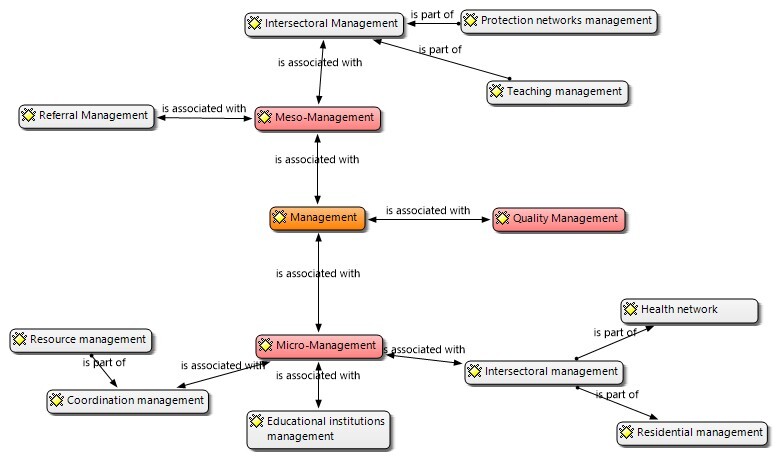
Clinical management—meso-level management, quality management, and micro-level management

*Note: Figure created using ATLAS.ti software.*

Figure 1 shows the three main meta-categories related to "Care and Management": meso-level, micro-level, and quality.

**1. Meso-level management**

Focused on the organization of healthcare networks, this meta-category corresponds to one of the levels of management associated with network managers, including healthcare services and the respective departments within the healthcare network. At the meso level, management activities include supervision, support from technical advisors, resource management, and coordination with the intersectoral network (including health networks, protection networks, and social programs, among others). Two subcategories can be identified: intersectoral care and care provided following referral.

[I can connect with universities and other settings; for example, when we need to discuss referral pathways, we can hold meetings with other technical advisors or with other institutions and centers like CESAMCO, the adult day hospital. I can help coordinate with the referring hospital, but the key is that this shouldn’t interfere with the day hospital’s own management processes…] I9 US15

**1.1. Inter-sectoral care:** There is support and supervision of goals; however, the hospitals under study lack an updated management model, with the most recent version dating back to 2001, which hinders effective management, particularly in the allocation of human and material resources.

[Centers are managed by providing administrative support to the teams. This administrative work may include clinical care; it must be supervised to ensure compliance with the ministry’s goals; policies related to the area must be communicated to the personnel; professionals must be monitored; and any difficulties, such as sick leave or time off, must be addressed…] I9 US8

**1.2. Care following referral:** Focused on support, ongoing communication, and coordination, while fostering participatory initiatives. It also involves advocating for hospital-related issues with management areas (clinical cases, human resources, supplies) to find solutions. The area requiring most intervention is the judicial sector, as legal cases or those involving residential care systems require greater management and coordination with protection network agents.

[The technical advisor’s perspective is necessary when cases reach a standstill or when the network needs to coordinate efforts; sometimes teams get stuck, and the technical advisor has to move things forward… and therapists should also have therapist supervisors… or when you’ve achieved a level objective] I11 US2

**2. Micro-level management**

Focused on the organization of healthcare networks, this meta-category links one of the management levels in relation to clinical management, which encompasses direct care and treatment provided by healthcare teams. At this level, efforts made include coordination, intersectoral collaboration, school management, community-based initiatives, and resource management (both human and material).

**2.1. Coordination:** Within the role of the CADH coordinator, this function serves as a link between meso-level and participating entities, facilitating the flow of directives and participating in thematic working groups to ensure that needs are met. Resources (human and material), financial resources, pharmacy supplies, and quality control are managed by a nurse and the coordination team.

[To manage human and material resources to ensure the hospital runs properly... to coordinate everything with external institutions, including referrals both internal and external, manage capacity and staff, and fulfill all our roles.] I3 US36

**2.2. Intersectoral collaboration:** Coordination efforts are conducted with CESFAM, the polyclinic, and CESAMCO, as well as with the protection network and the Child and Adolescent Protection Service (formerly “SENAME” or “Mejor Niñez”). This is one of the most complex networks, due to limited awareness among judicial teams of the mental health process in children and adolescents, as well as a lack of personalized care in residential care facilities, which contrasts with the care recommended and provided by the CADH’s professional team.

[Children in residential care go through long processes, and many times these are not therapeutic discharges…andinterventionprocessesaremoresuccessfulwhenchildrenhaveafamilyenvironment… and a support network. When that’s not there, it’s very difficult to move forward] I1 US 35

[The high staff turnover at “Mejor Niñez” makes our work very difficult, because the children’s key caregivers keep changing, coordination efforts are left unfinished, and we have to start over. Some practices are questionable from the institutional perspective of “Mejor Niñez”, which also influences professional practice… It’s a very complex situation when patients come from residential care facilities; it’s a challenge for us as a hospital] I3 US42

**2.3. Education:** Within school-related management, direct communication is maintained with the educational institutions attended by the children and adolescents enrolled in the CADH. The educational psychologist, occupational therapist, psychologist, and nurse play a key role by providing active, personalized psychoeducation to teachers and personnel on how to interact with patients. During the first stage of treatment, patients do not attend school in order to focus on clinical care and stabilize the acute phase. There are also challenges in understanding these cases within educational institutions, such as discrimination and rejection toward children and adolescents in the acute phase of mental health.

[We need to raise awareness about mental health and develop management strategies without resorting to exclusion, which is often the case due to a lack of knowledge and the absence of crisis management strategies, or when the condition becomes acute, the tendency is to exclude. We need to keep bringing the issue of mental health to the forefront to prevent stigmatization and discrimination] I1US12

[Coordination meetings are held with schools, and curriculum adjustments are made to better manage and organize students for their gradual reintegration into school settings. Many of the students who face, or have faced, difficulties with school integration do so because of their mental health issues] I6 US28

**2.4. Community context:** Clinical professionals organize activities aimed at promoting the social integration of patients through community-based initiatives, such as trips to museums and parks, connections with universities, youth centers, and cultural programs, among others. It is worth noting that these activities are not part of the hospital’s formal program; rather, they are initiated by the participants themselves and are therefore self-managed.

[We’re constantly striving to connect with the community so that teenagers have the opportunity to learn about the various activities and opportunities available in the city, with the aim of broadening their areas of interest a bit] I7

**3. Quality of care:**

The quality unit is led by a nurse who develops protocols tailored to the hospital's needs (e.g., suicide risk, psychomotor agitation, and medication administration), ensures patient safety, and oversees the delivery of quality care during the intervention. It is also where adverse events are reported, leading to the implementation of continuous improvement measures; however, there is a lack, or absence, of a flowchart to address these needs with management, which limits the development of strategies to improve the facility. This is linked to the fact that, at the meso-level, there is no quality unit within the Mental Health Department to oversee and generate quality guidelines spanning across all mental health facilities. An important aspect of quality assurance is supporting the application for health authority approval in tandem with technical management areas, an initiative that has been underway for years; however, it has not yet been successful due to bureaucratic difficulties involving the Mental Health Department and infrastructure.

[We're working toward obtaining health authority approval; we've been trying since 2018, but it hasn't worked out for various reasons…we have to go to the CESAMCO pharmacy to pick up our controlled substances] I5US57

[The nurse plays a key role in this and is responsible for implementing and ensuring compliance with local protocols, verifying that quality indicators are met… reporting all sentinel events… and putting together a work plan to prevent them from happening again. They’re also in charge of the pharmacy unit, ensuring that pharmacy protocols are followed, that medications are delivered on time, and that there are no adverse effects in either dispensing or administrating] I7

**Table 1:** Challenges related to care for children and adolescents with acute mental health conditions

Challenges related to care	Awareness-raising Public policies Undergraduate education	Community education Improvements in resources, the availability of specialized mental health centers, and in policies or guidelines. Incorporation

**4. Challenges related to care**

Awareness of these types of healthcare facilities is identified as a key need, promoted through outreach, visibility, and education, fostering recognition across different networks and within the community. Improving public policies focused on current needs at the national and regional levels, and refining guidelines, will directly influence organizational structure and ensure adequate resource allocation. Under this same framework, increasing the number of specialized mental health centers will reduce gaps or barriers, enabling high-quality care commensurate with the complexity of cases, preventing burnout among existing teams, and redirecting resources away from strategies that do not yield solutions. A key challenge is the need to increase undergraduate training in mental health and its application in acute care settings; this would foster greater willingness and internalization when providing care, learning from experience while building on a clear, concise educational foundation, which is essential for the management of these healthcare facilities.

[This issue needs to be made more visible… to reduce gaps in team interventions, structural gaps that need to be addressed, to create better working conditions and support the stabilization of acute patients… and to prevent them from requiring 24/7 hospitalization, allowing them to be integrated into a stable family or residential care facility] I5 US79

[How can we keep moving forward with the implementation, dissemination, and refinement of the model and establish a presence in professional training settings… so that there are more experienced professionals who can continue advancing its implementation to other locations?] I6 US15

[Mental health is like the neglected sibling of health, and child and adolescent mental health is like the neglected sibling of that neglected sibling, or perhaps the neglected child of the neglected sibling of health. And in that sense, I think we need more facilities of this kind] I6 US72

The integration of Edgar Morin’s Theory of Complex Thought adds value by addressing contemporary and emerging challenges that require a complex approach, with interventions targeting both individuals and organizational structures. Similarly, Patricia Benner’s philosophy allows for the identification and categorization of professionals as competent and efficient, prioritizing planning and management, thereby increasing efficiency in working with children and families^37^-^39^.

## Discussion

Regarding intersectoral care and deficits in the management model, one study reports challenges in program implementation, quality management, and deinstitutionalization, as well as deficits in standardized procedures and tools for crisis management (aggression, suicide attempts, prevention and management of child abandonment, discharge management, and referrals)^57^.

Meanwhile, regarding micro-level management, the complexity of intersectoral coordination and management is evident. Most residential care facilities lack healthcare personnel, with no nurses available, resulting in deficits in supervision, training, and the development of crisis intervention strategies. As direct-care educators, nurses are responsible for the pharmacological area; their absence leads to coordination gaps manifested as delays, lack of engagement, or lack of understanding, along with high personnel turnover. These factors limit care for children and adolescents, including the provision of clinical medical information regarding mental health care^58^. Along the same lines, a manual of nursing procedures in community mental health addresses strategic planning, clinical management, and care plans, describing operational procedures and standards of care aimed at ensuring continuity of care^59^.

One study highlights the complexity of these conditions and the therapeutic approach, which entails multidisciplinary, multimodal, and coordinated care, an approach that is essential for serious conditions such as psychosis, a finding that aligns with the present study’s results^60^. In the educational context, the measures described affect individuals with mental health issues who are vulnerable and experience stigma, discrimination, abuse, and violence, and who may be excluded from educational and employment opportunities. This is consistent with the stigma faced by children and adolescents, which leads to limited acceptance and difficulties in their social, community, and school reintegration^61^.

Regarding quality, a study highlights its key role in mental health care, guiding efforts to improve effectiveness. Although this aspect follows similar guidelines, delays in implementation strategies are evident, as it remains an area that is insufficiently recognized. This is consistent with the present findings, as evidenced by the lack of health authority approval and deficiencies in protocols spanning across the region’s facilities^62^.

Among the challenges, expanding mental health coverage for children in Chile underscores the need to prioritize child mental health in order to prevent early life experiences from having a significant impact on brain development. Investments in promotion, prevention, and early treatment are cost-effective and efficient and represent the most efficient and equitable strategy for human capital development^63^. The International Council of Nurses emphasizes the essential role of mental health research in addressing disparities in access to care and improving the effectiveness of care, thereby promoting the development of policies that enhance equitable services and access. This aligns with the overarching category of challenges related to care^64^.

Undergraduate education, along with postgraduate specialization programs or master’s degrees in mental health, is essential for reducing stigma and understanding the complexities of children experiencing emotional distress^65^.

Regarding challenges and professional roles, the authors propose a role-based theory of mental health, emphasizing comprehensive care and promoting proactivity, motivation, and self-management capacities^65^,^66^.

Finally, the population under study is characterized by its vulnerability. A large proportion of children and adolescents experience socioeconomic difficulties, histories of abuse, domestic violence, limited support networks, highly burdened caregivers, a history of substance use within the close social environment, experiences of complex trauma, and involvement in residential care facilities, among other factors. Consequently, one of their defining characteristics is complexity, which can also pose an obstacle to care. In this context, rights violations and sociocultural deficits function as triggering factors among children and adolescents enrolled in child protection programs, leading to increased administrative and meso-level management tasks that require specialized professionals.

## Conclusions

The study objective was achieved, showing that the care provided in Child and Adolescent Day Hospitals (CADH) is shaped by the management of professional teams and the implementation of preventive measures, even among a highly vulnerable and socially complex population. The planned methodology was successfully implemented, enabling the collection and analysis of results.

Child and Adolescent Day Hospitals play a key role in addressing the specific needs of their target population, in a context marked by growing demand for child and adolescent mental health care and system fragility. This requires government institutions to implement strategies aimed at reducing waiting lists, promoting continuity of care, decreasing relapse rates, and strengthening social rehabilitation. In this context, work with families and clinical management is crucial, with particular emphasis on the contribution of nursing in collaboration with multidisciplinary teams, as they coordinate the different dimensions of care necessary to achieve therapeutic goals.

Likewise, the importance of micro-level and meso-level management in care is highlighted, as these are areas where teams’ efforts are often not sufficiently recognized. Strengthening internal quality processes would support the development of a culture of safety, bridge gaps, and consolidate specialized quality management units, a field that remains underdeveloped in mental health. In this regard, nursing professionals possess comprehensive competencies to lead such implementation and guide continuous improvement. Alongside this, it is necessary to address the depersonalization of care in residential care facilities through adequate resources, clear protocols, continuous training, and access to mental health specialties.

Finally, government institutions, administrators, and authorities are urged to recognize the existing gaps in the mental health network, prioritizing evidence-based public policies that meet current demands. This would have a positive impact on public health, resource allocation, and the professional practice of clinical teams.

In terms of limitations, this study focused on a single facility in the southern region and did not include other centers within the national network. Therefore, future research should be expanded to different contexts and employ various methodological designs to strengthen the disciplinary foundation in child and adolescent mental health and generate more robust evidence regarding the care provided.
